# Supplementary material for: Structural Modification and Optimisation of Hyperoside Oriented to Inhibit TGF-β-Induced EMT Activity in Alveolar Epithelial Cells
Source: Pharmaceuticals (Basel). 2024 May 2;17(5):584. doi: 10.3390/ph17050584 (PMC11124421; doi:10.3390/ph17050584)
Supplement: Supplementary file 1 [file pharmaceuticals-17-00584-s001.zip › pharmaceuticals-2948303--supplementary.pdf]

Supplementary Material

Structural Modification and Optimization of Hyperoside Oriented to Inhibit TGF-β Induced EMT Activity in Alveolar Epithelial Cells

Ziye Gao [1,†], Mengzhen Xu [1,†], Chuanguo Liu [2], Kai Gong [1], Xin Yu [1], Kaihui Lu [1], Jiang Zhu [1], Haixing Guan [3,*] and Qingjun Zhu [1,4,*]

1 Innovative Institute of Chinese Medicine and Pharmacy, Shandong University of Traditional Chinese Medicine, Jinan 250355, China; 2021111377@sdutcm.edu.cn (Z.G.); 2023100144@sdutcm.edu.cn (M.X.); 2022111508@sdutcm.edu.cn (K.G.); 2022110141@sdutcm.edu.cn (X.Y.); 2022111509@sdutcm.edu.cn (K.L.); 2023111502@sdutcm.edu.cn (J.Z.)

2 Experimental Center, Shandong University of Traditional Chinese Medicine, Jinan 250355, China; 60011973@sdutcm.edu.cn

3 Experimental Center, Shandong Provincial Key Laboratory of Traditional Chinese Medicine, Key Laboratory of Traditional Chinese Medicine Classical Theory, Ministry of Education, Shandong University of Traditional Chinese Medicine, Jinan 250355, China

4 Key Laboratory of Traditional Chinese Medicine Classical Theory, Ministry of Education, Shandong University of Traditional Chinese Medicine, Jinan 250355, China

* Correspondence: haixingguan@163.com (H.G.); zhuqingjuncn@hotmail.com (Q.Z.)

† These authors contributed equally to this work.

Experimental procedures and characterisation of all new compounds including $_{1}$H, $_{13}$CNMR

Table and Figure Caption

Figure S1.^{1}H NMR spectra of compound **G0**

Figure S2. ^{13}C NMR spectra of compound **G0**

Figure S3.^{1}H NMR spectra of compound **G1**

Figure S4. ^{13}C NMR spectra of compound **G1**

Figure S5.^{1}H NMR spectra of compound **G2**

Figure S6. ^{13}C NMR spectra of compound **G2**

Figure S7.^{1}H NMR spectra of compound **G3**

Figure S8. ^{13}C NMR spectra of compound **G3**

Figure S9.^{1}H NMR spectra of compound **G4**

Figure S10. ^{13}C NMR spectra of compound **G4**

Figure S11.^{1}H NMR spectra of compound **G5**

Figure S12. ^{13}C NMR spectra of compound **G5**

Figure S13.^{1}H NMR spectra of compound **G6**

Figure S14. ^{13}C NMR spectra of compound **G6**

Figure S15.^{1}H NMR spectra of compound **G7**

Figure S16. ^{13}C NMR spectra of compound **G7**

Figure S17.^{1}H NMR spectra of compound **G8**

Figure S18. ^{13}C NMR spectra of compound **G8**

Figure S19.^{1}H NMR spectra of compound **G9**

Figure S20. ^{13}C NMR spectra of compound **G9**

Figure S21.^{1}H NMR spectra of compound **G10**

Figure S22. ^{13}C NMR spectra of compound **G10**

Figure S23.^{1}H NMR spectra of compound **Z1**

Figure S24. ^{13}C NMR spectra of compound **Z1**

Figure S25.^{1}H NMR spectra of compound **Z3**

Figure S26. ^{13}C NMR spectra of compound **Z3**

Figure S27.^{1}H NMR spectra of compound **Z5**

Figure S28. ^{13}C NMR spectra of compound **Z5**

Figure S29.^{1}H NMR spectra of compound **Z6**

Figure S30. ^{13}C NMR spectra of compound **Z6**

Figure S31.^{1}H NMR spectra of compound **Z7**

Figure S32. ^{13}C NMR spectra of compound **Z7**

Figure S33 Effect of hypericin and its derivatives 48h on the activity of A549 cells

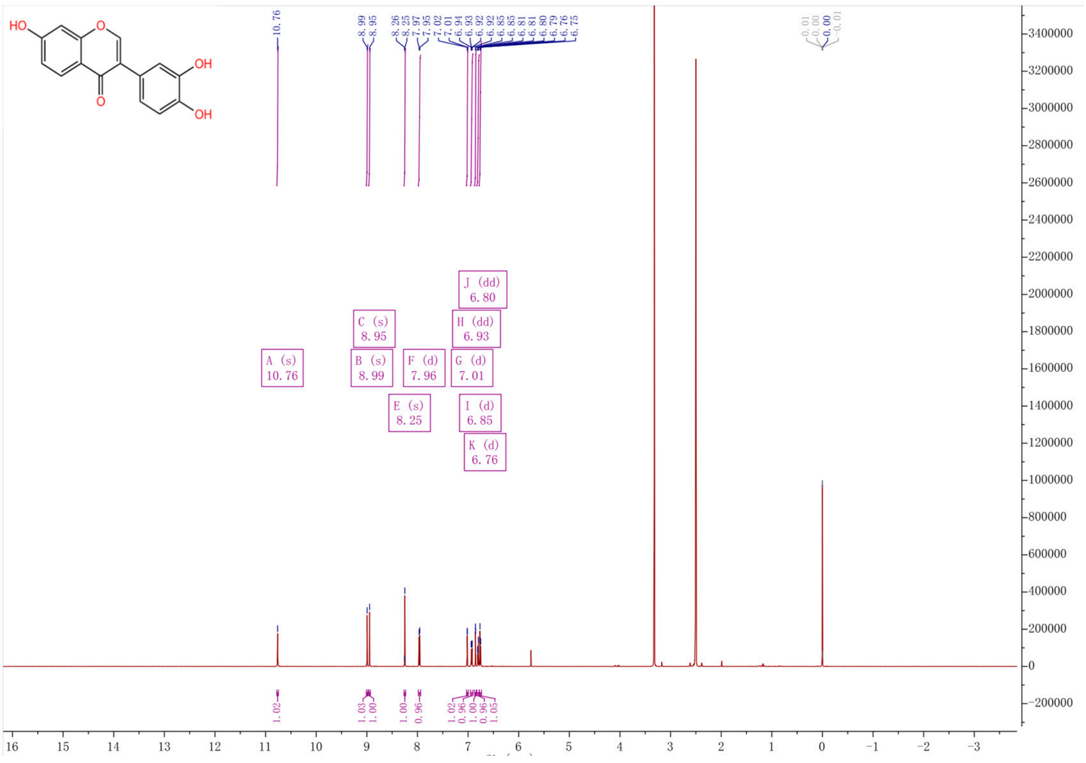

Figure S1.¹H NMR spectra of compound **G0**

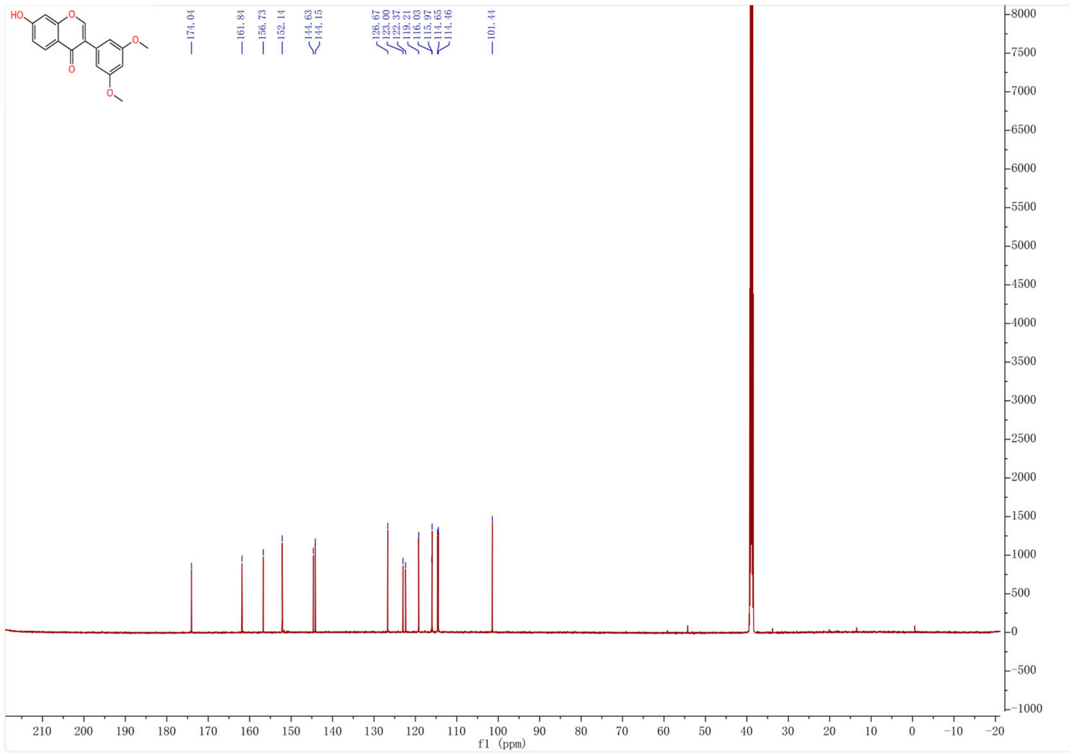

Figure S2. ¹³C NMR spectra of compound **G0**

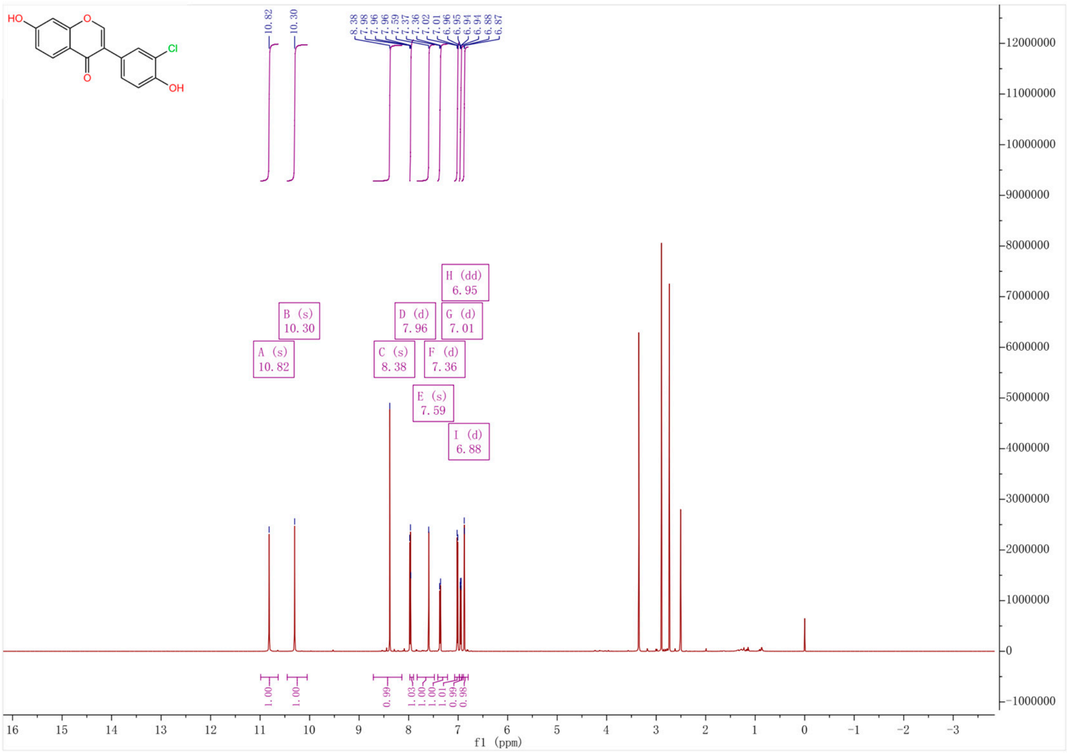

Figure S3.^{1}H NMR spectra of compound **G1**

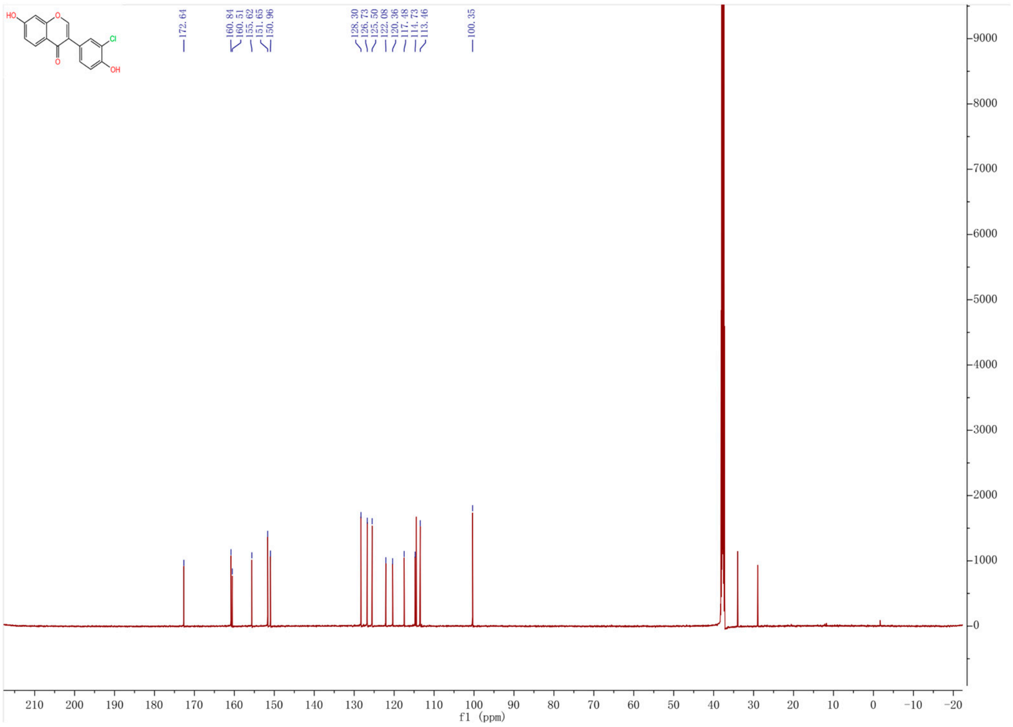

Figure S4. ^{13}C NMR spectra of compound **G1**

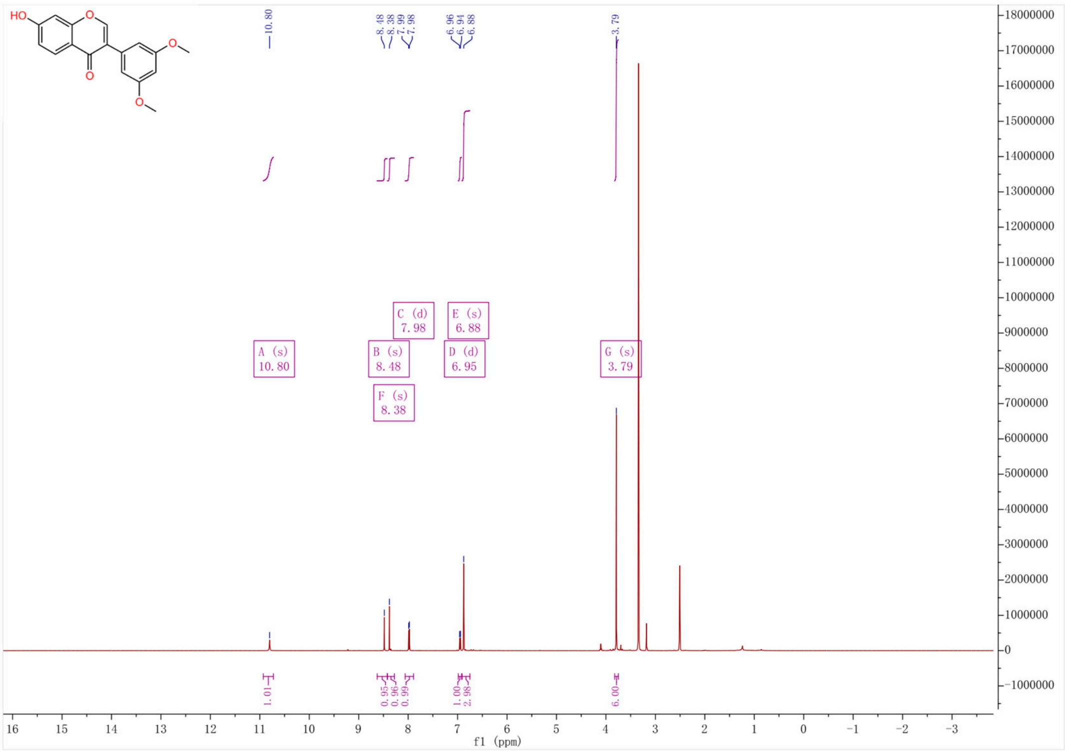

Figure S5.^{1}H NMR spectra of compound **G2**

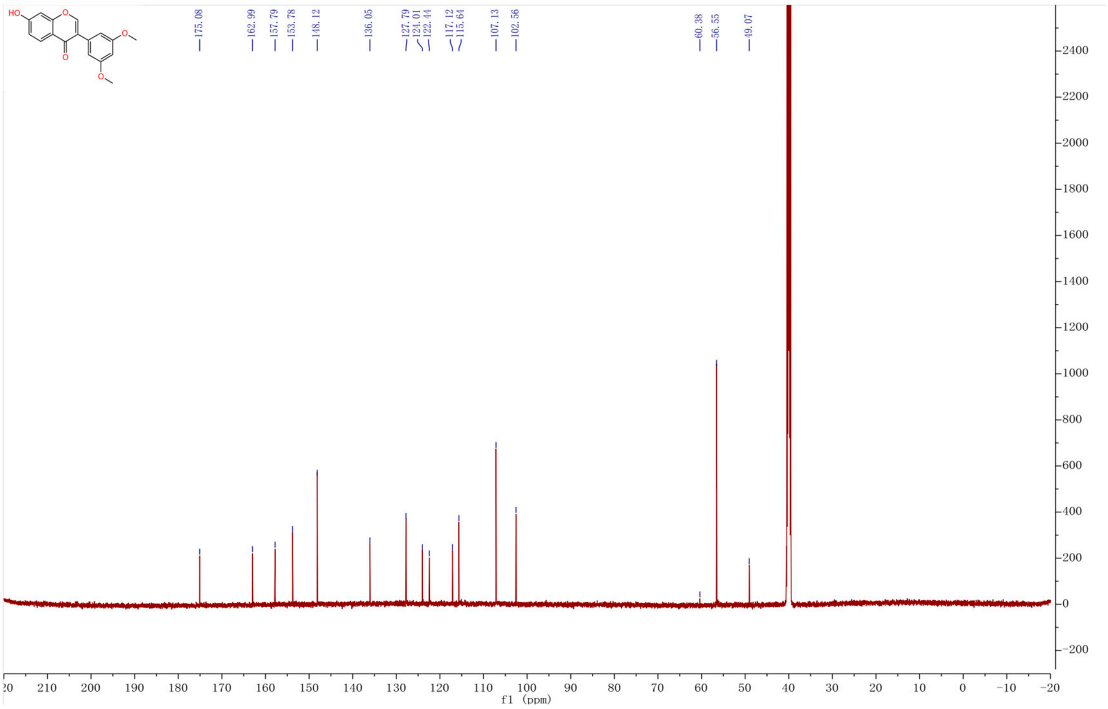

Figure S6. ^{13}C NMR spectra of compound **G2**

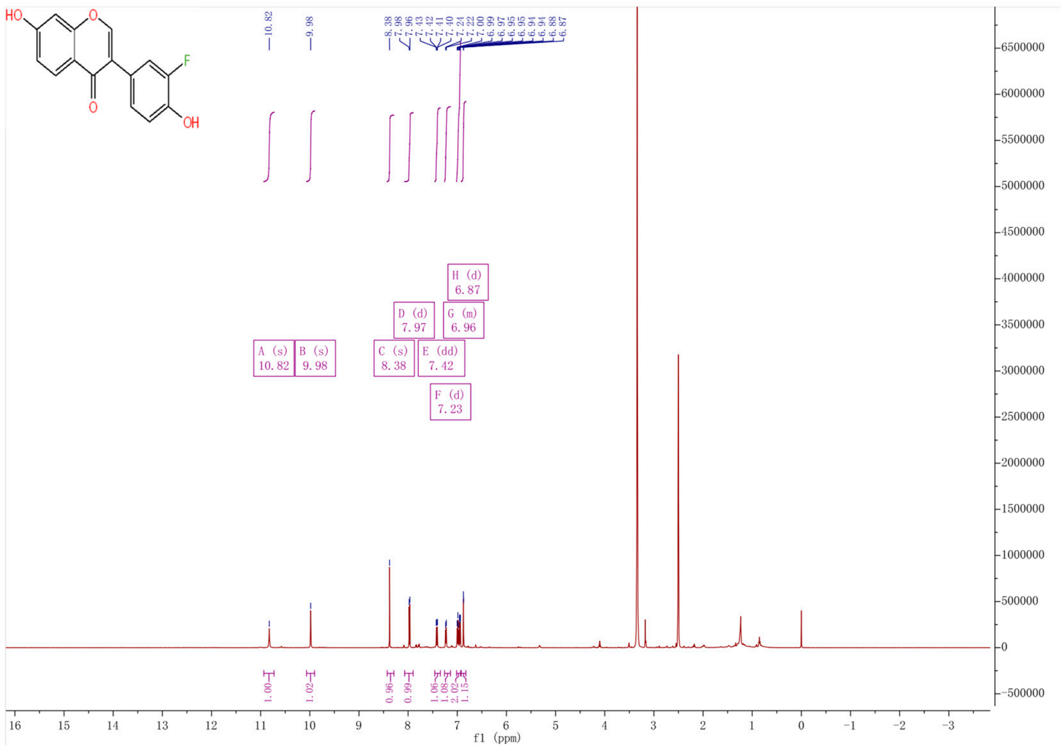

Figure S7.^{1}H NMR spectra of compound **G3**

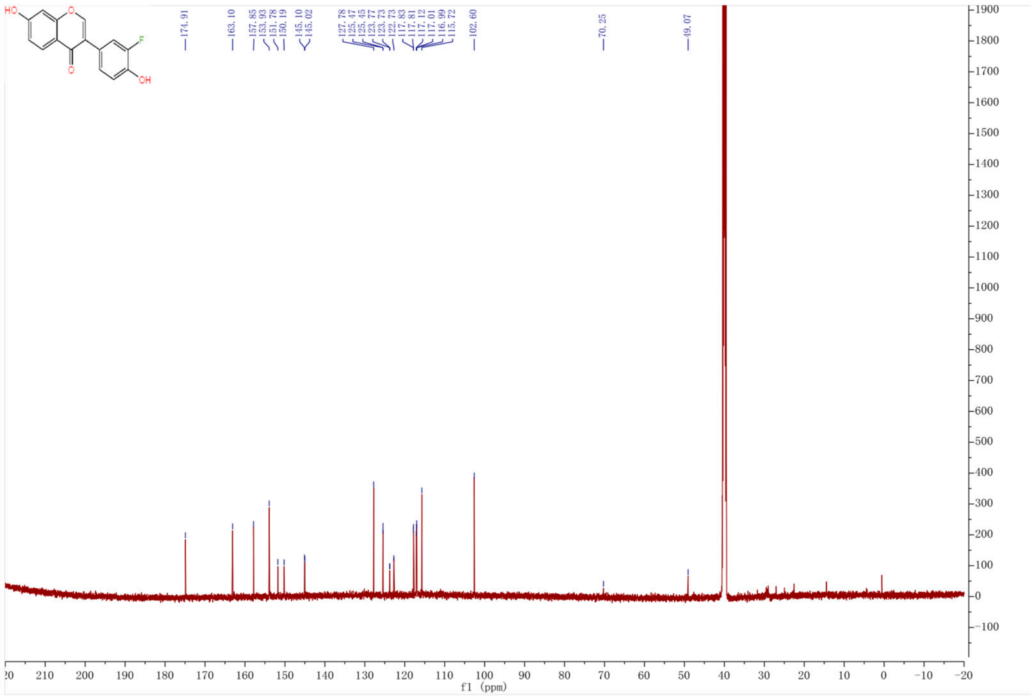

Figure S8. ^{13}C NMR spectra of compound **G3**

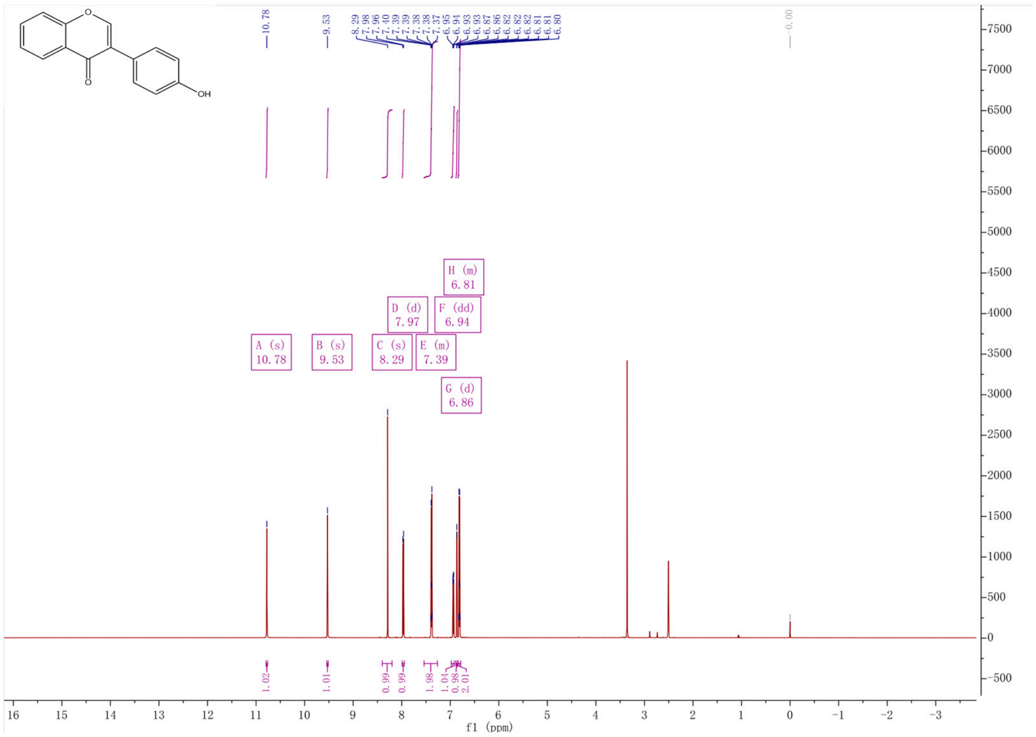

Figure S9.^{1}H NMR spectra of compound **G4**

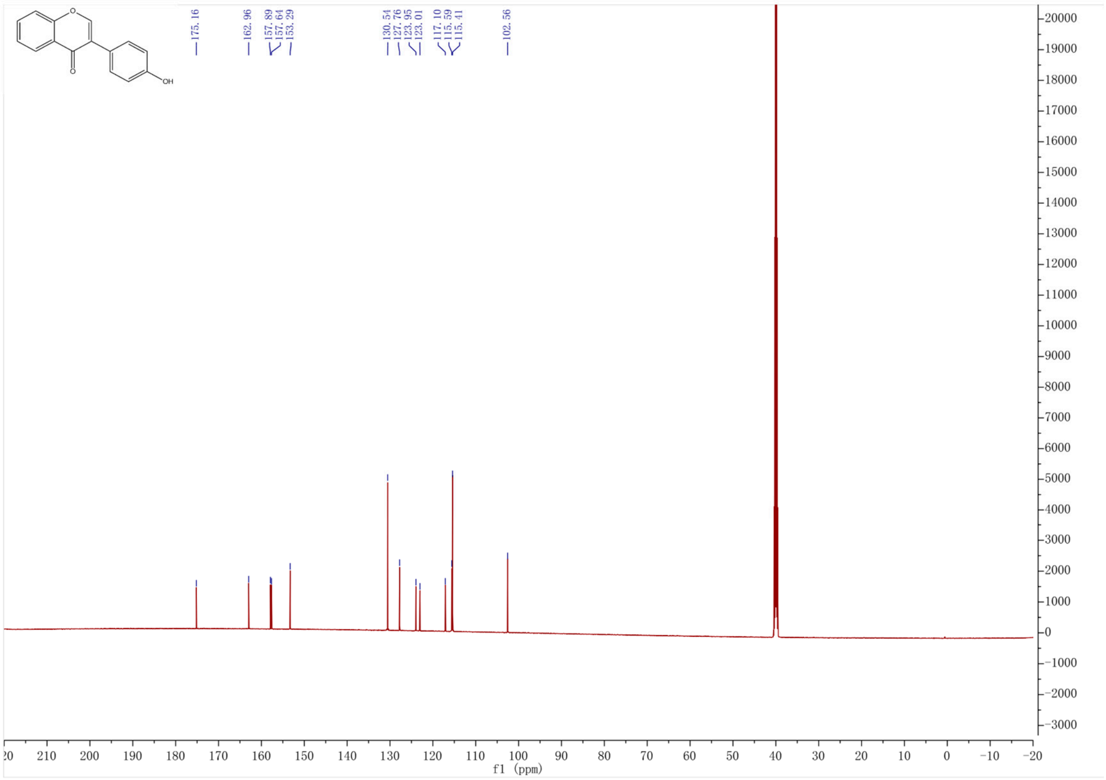

Figure S10. ^{13}C NMR spectra of compound **G4**

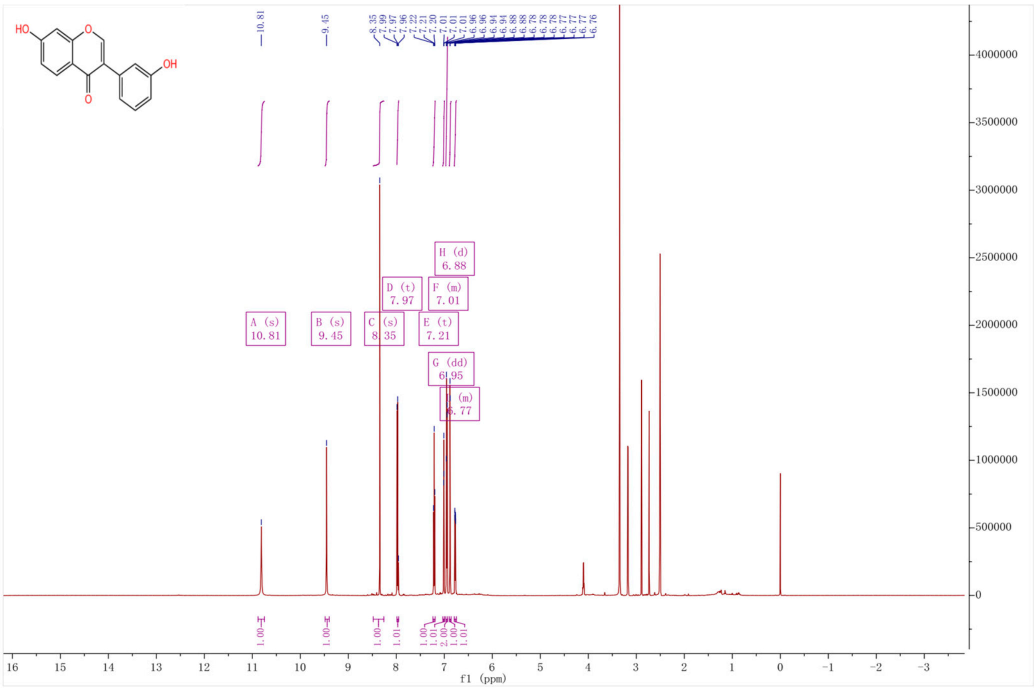

Figure S11.^{1}H NMR spectra of compound **G5**

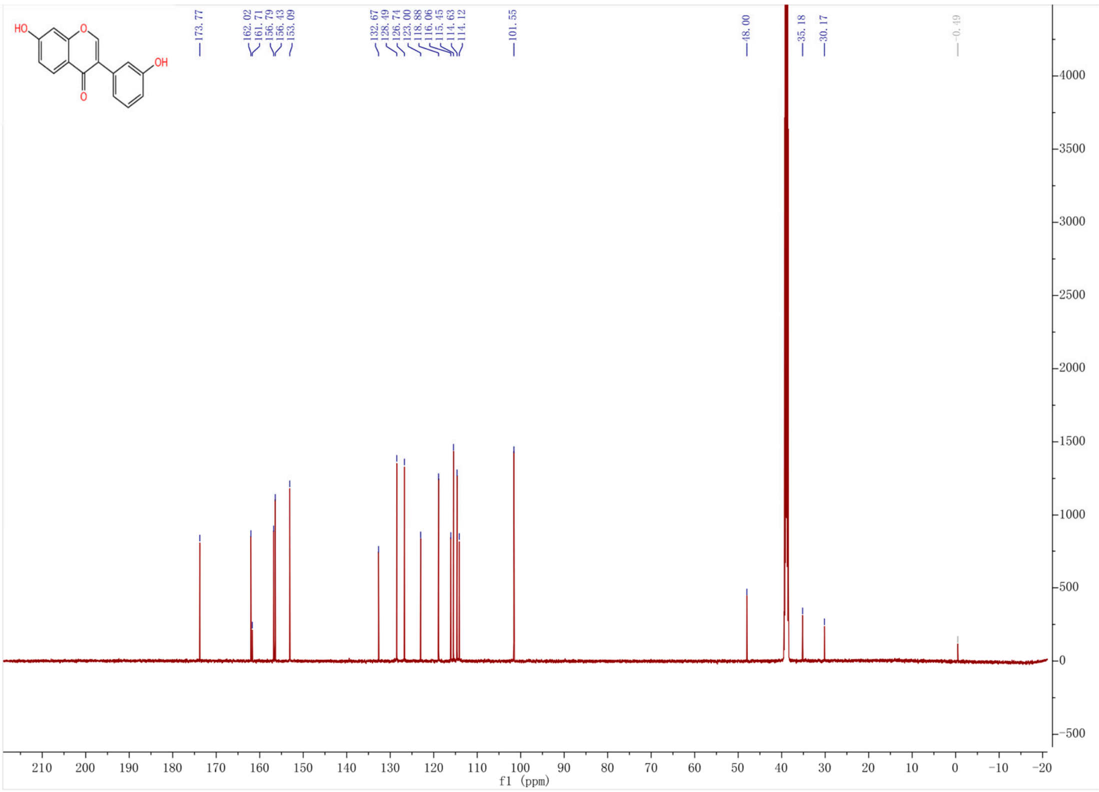

Figure S12. ^{13}C NMR spectra of compound **G5**

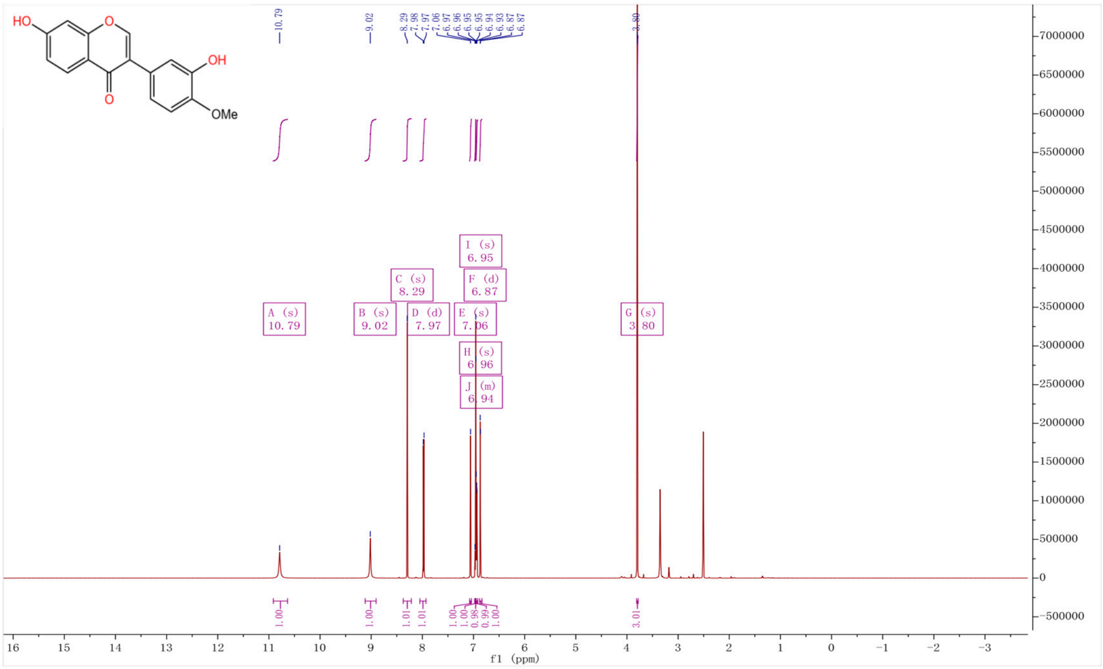

Figure S14. ^{13}C NMR spectra of compound **G6**

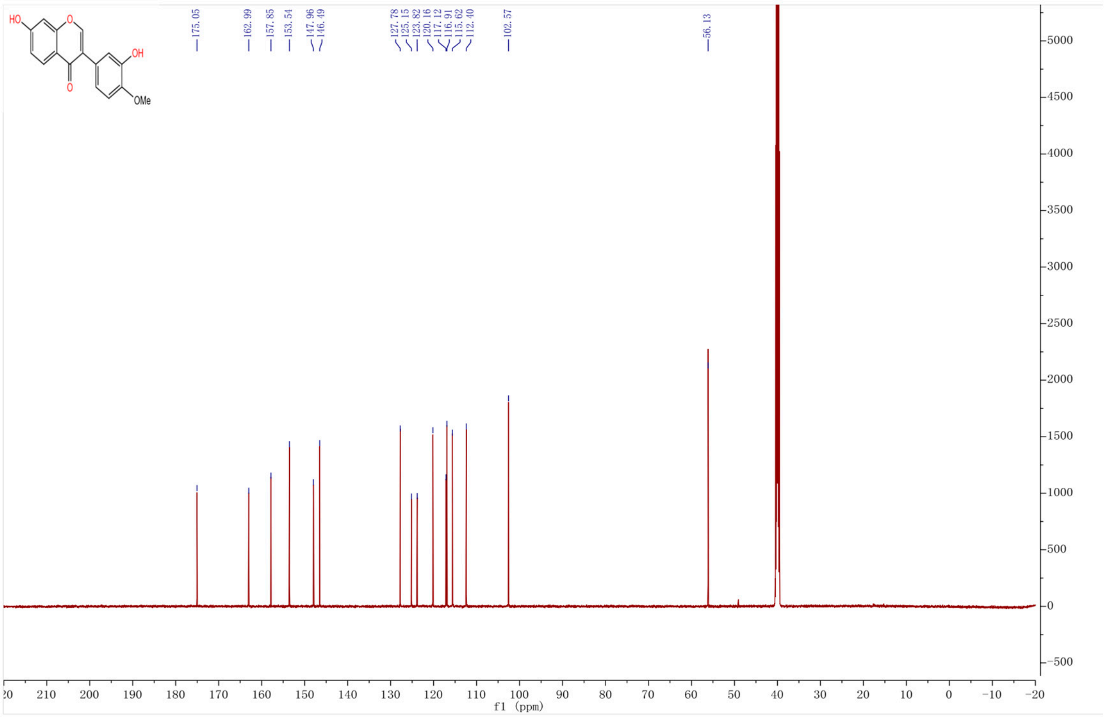

Figure S14. ^{13}C NMR spectra of compound **G6**

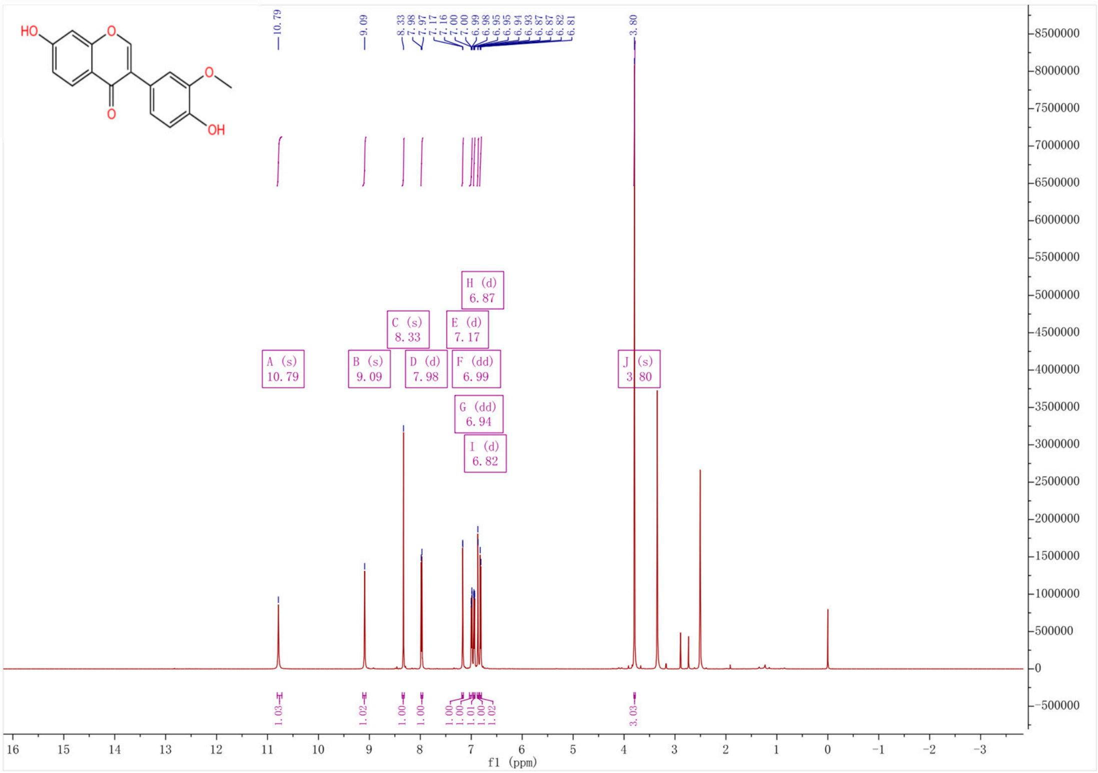

Figure S15.^{1}H NMR spectra of compound **G7**

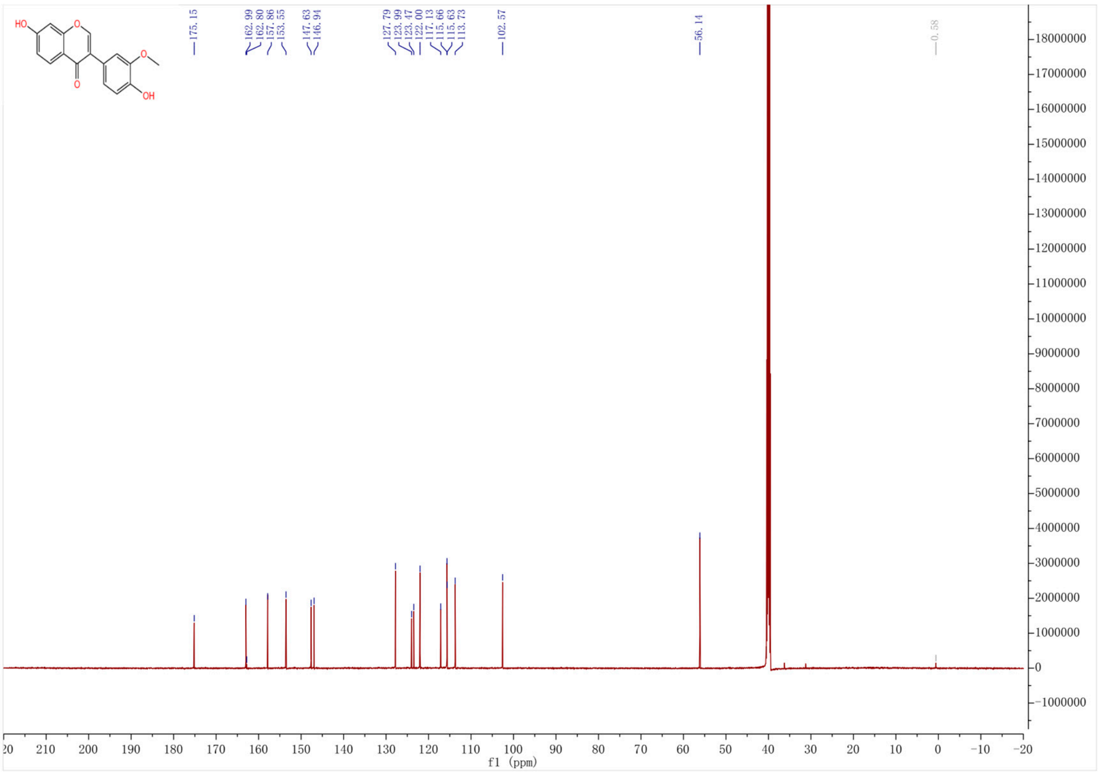

Figure S16. ^{13}C NMR spectra of compound **G7**

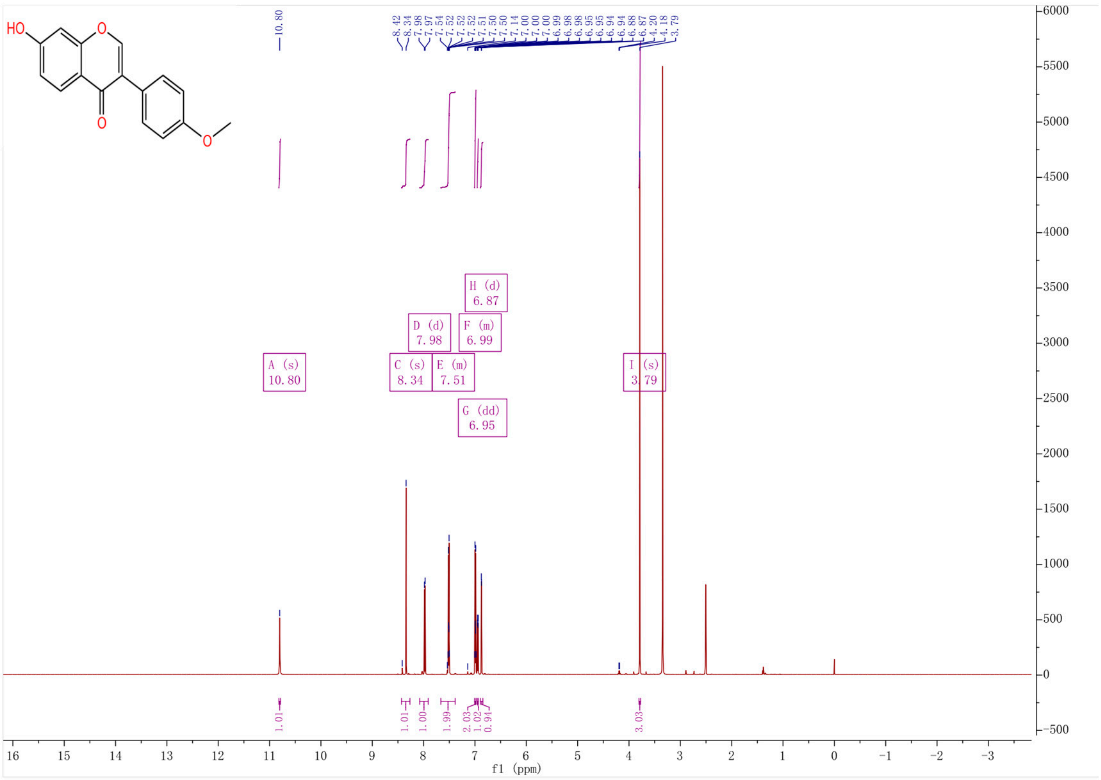

Figure S17.^{1}H NMR spectra of compound **G8**

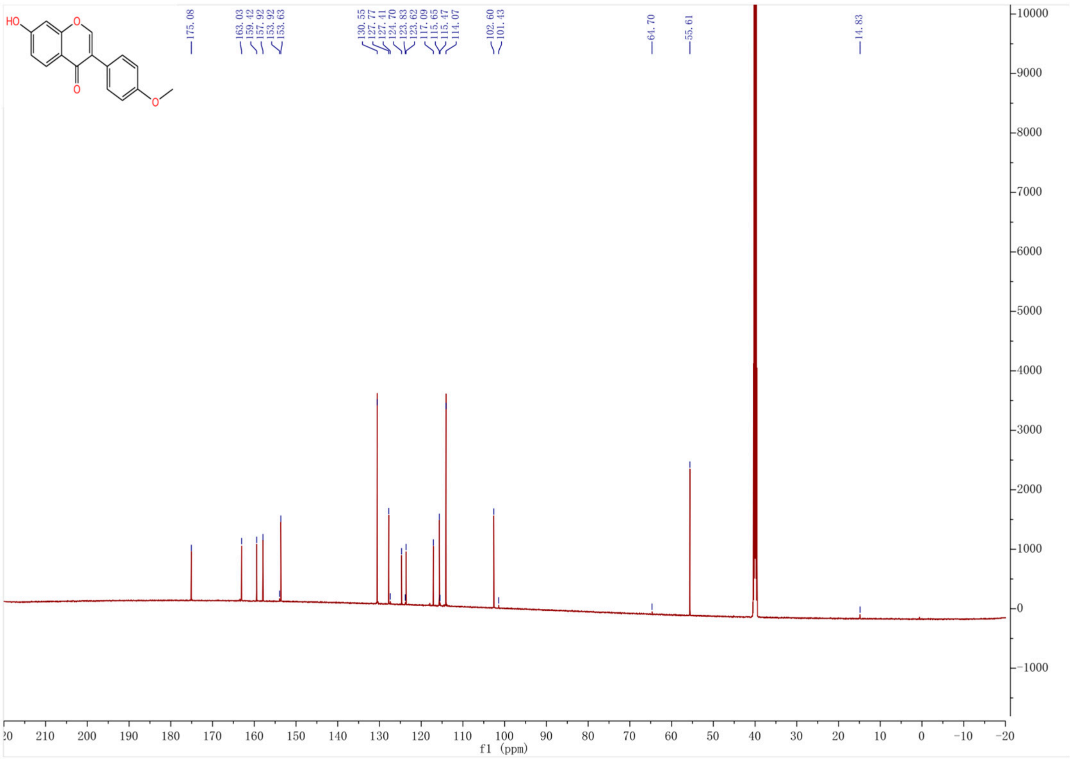

Figure S18. ^{13}C NMR spectra of compound **G8**

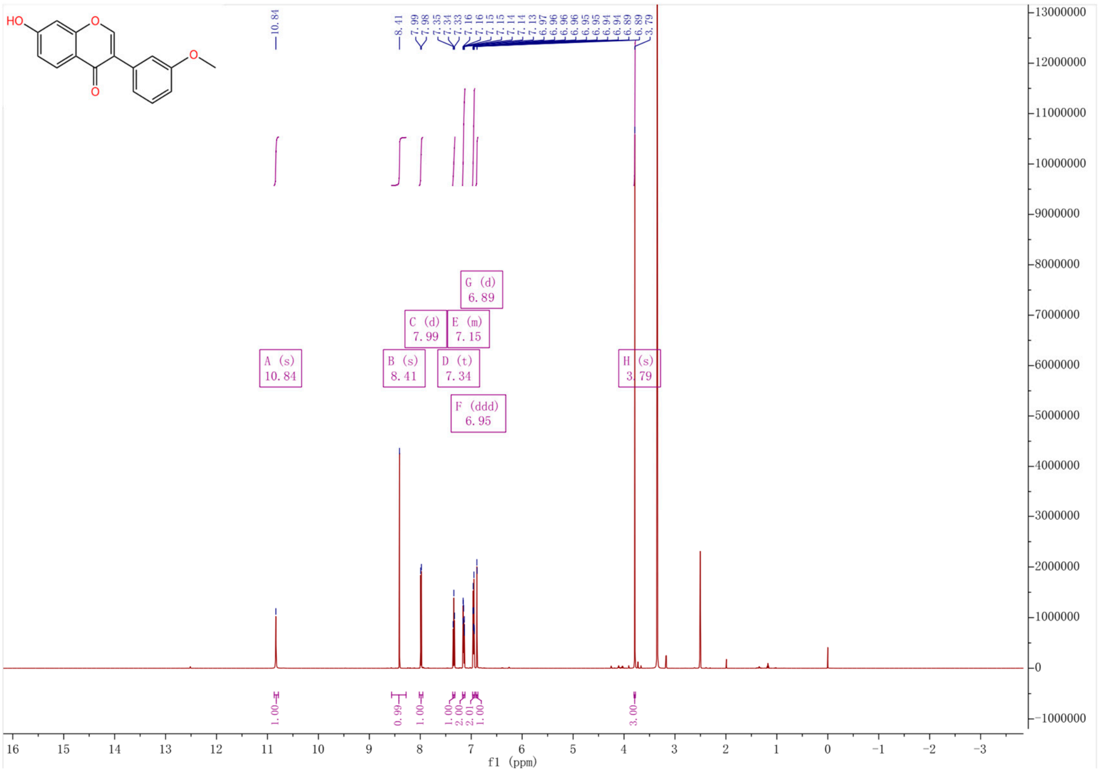

Figure S19.^{1}H NMR spectra of compound **G9**

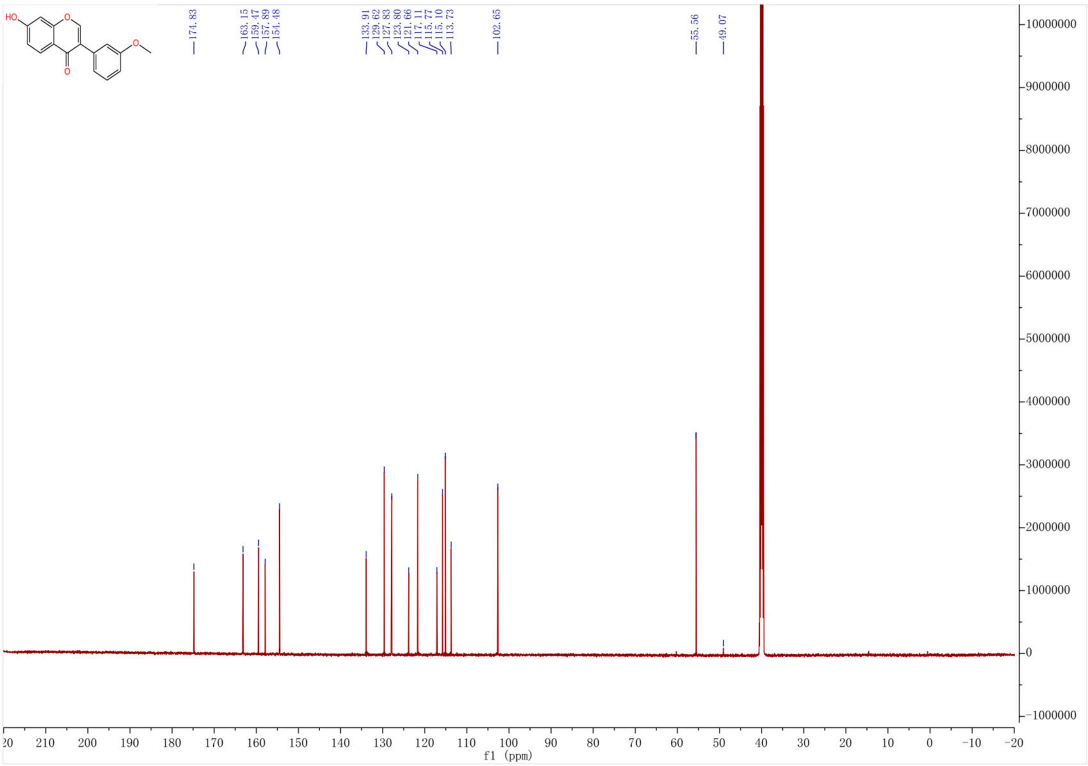

Figure S20. ^{13}C NMR spectra of compound **G9**

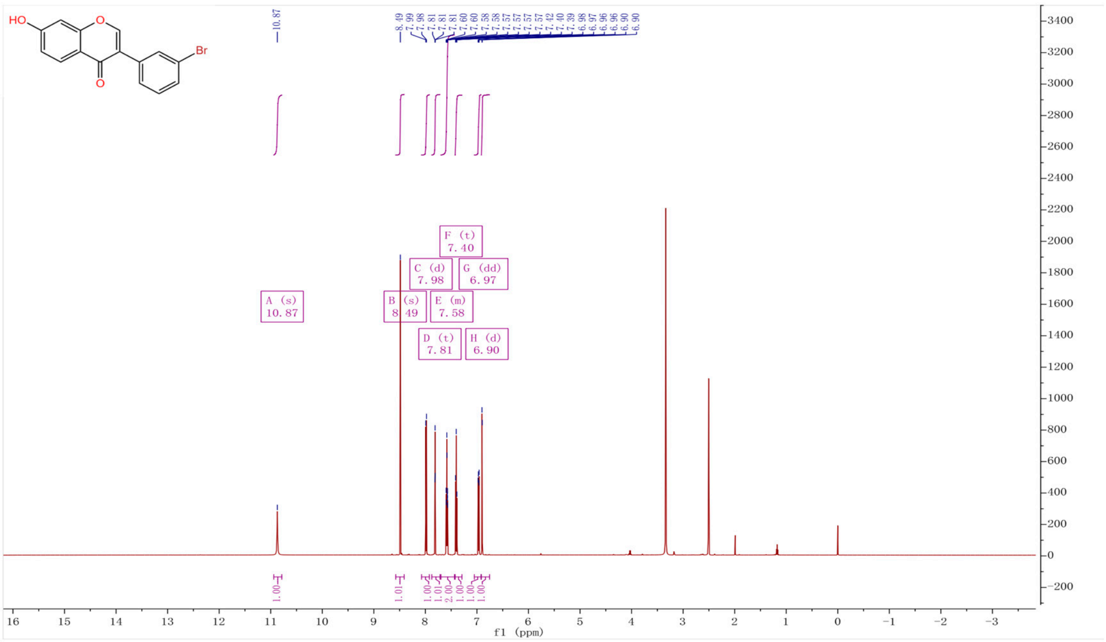

Figure S21.^{1}H NMR spectra of compound **G10**

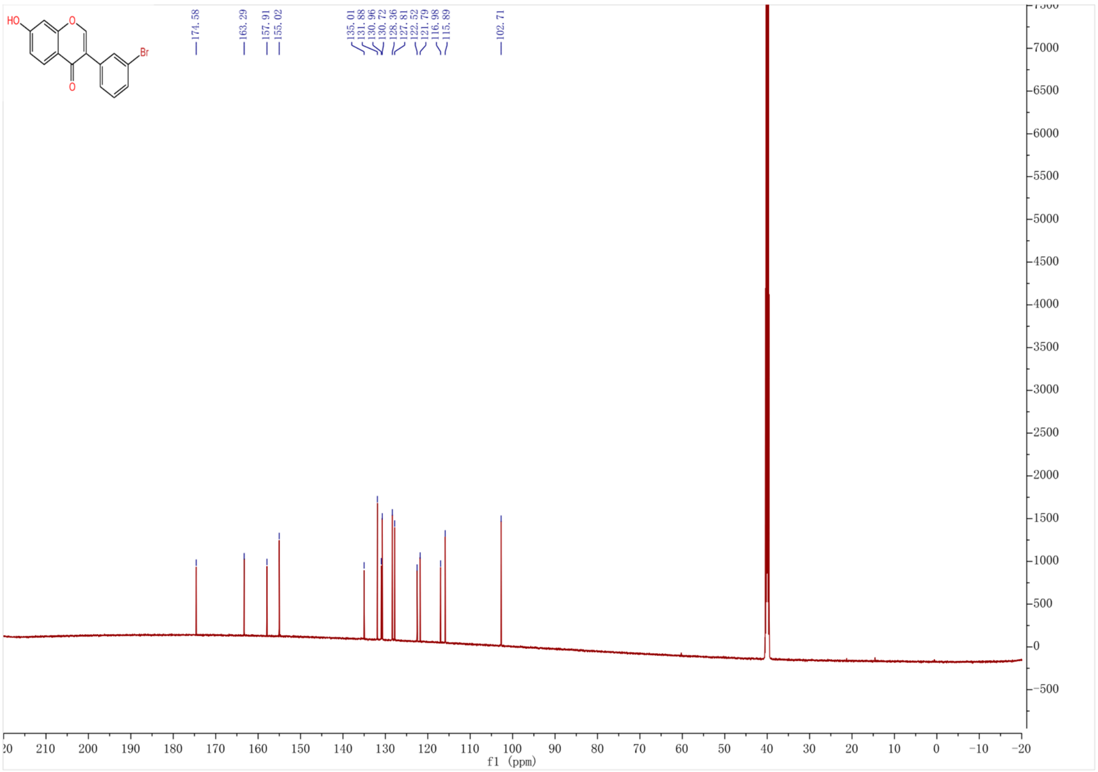

Figure S22. ^{13}C NMR spectra of compound **G10**

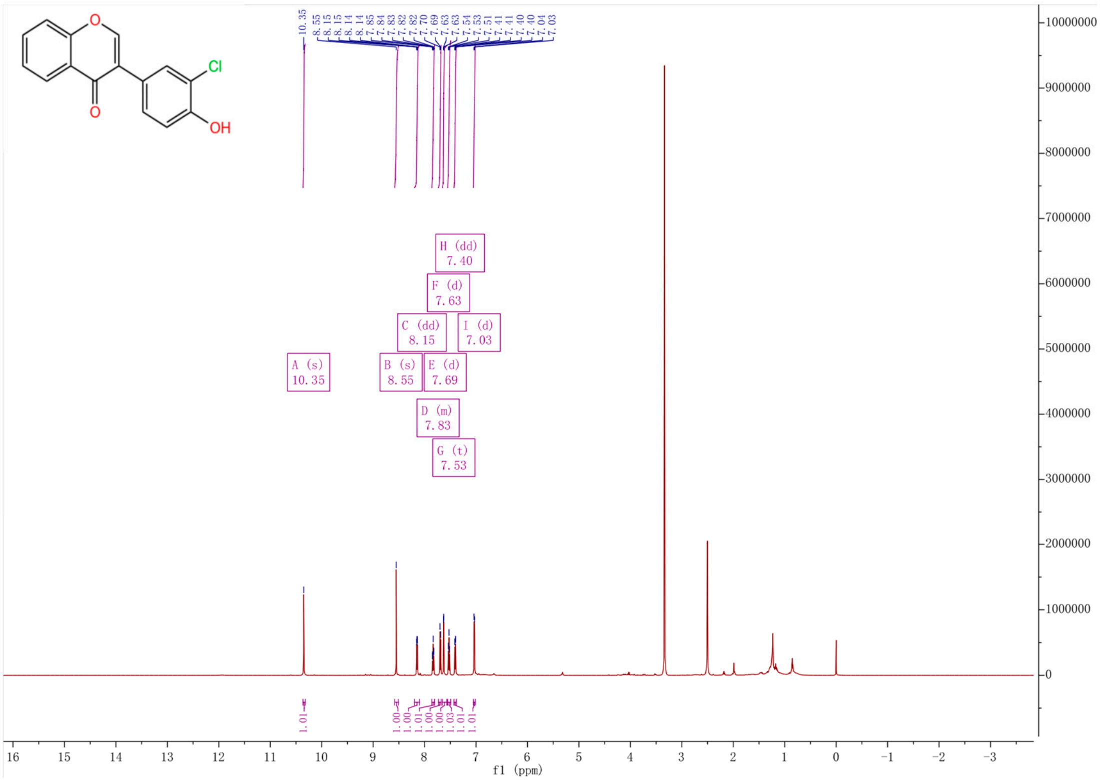

Figure S23.^{1}H NMR spectra of compound **Z1**

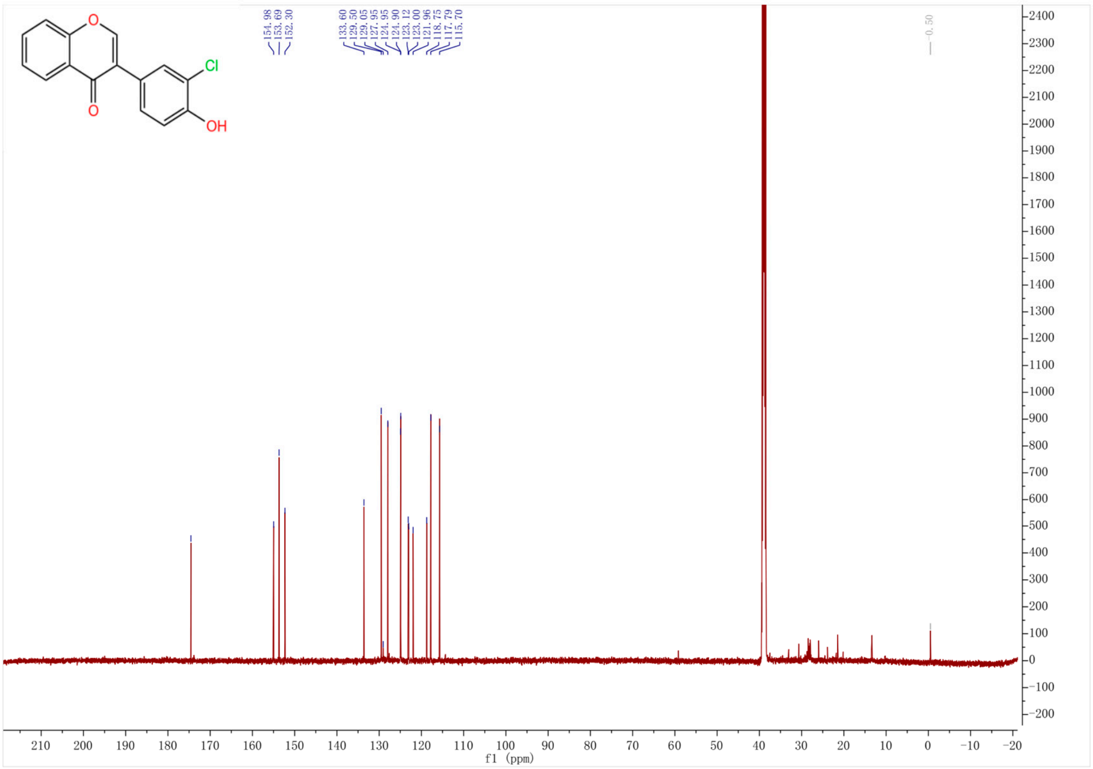

Figure S24. ^{13}C NMR spectra of compound **Z1**

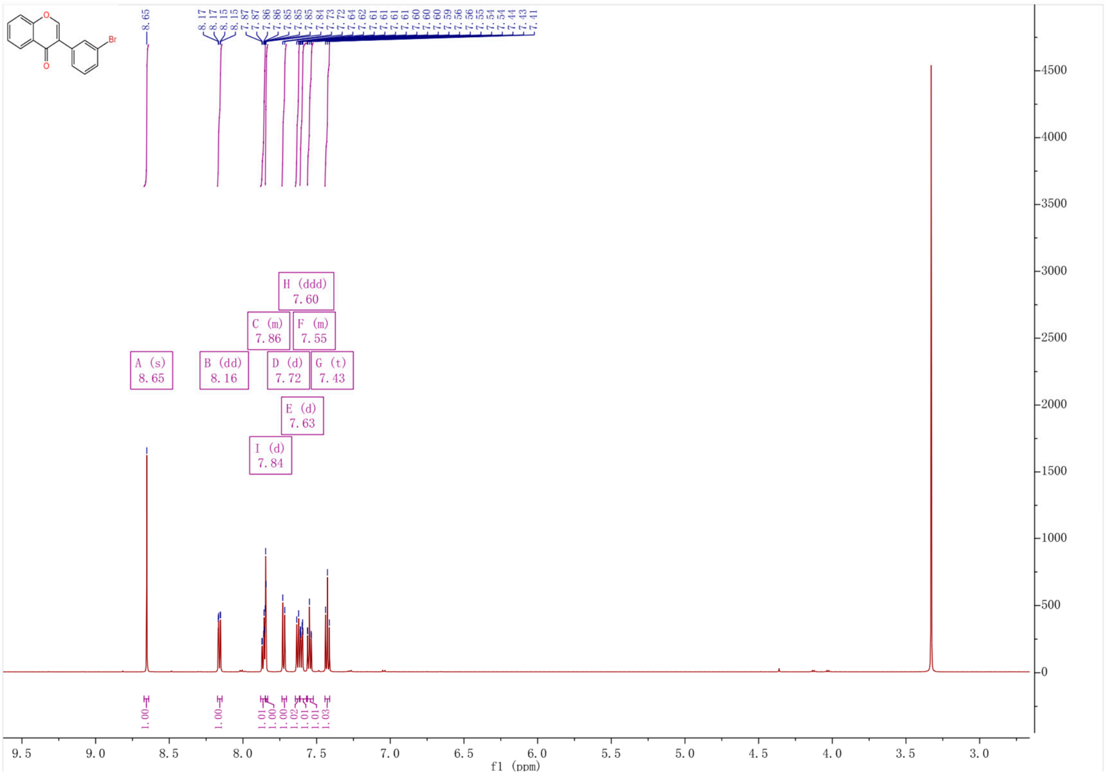

Figure S25.^{1}H NMR spectra of compound **Z3**

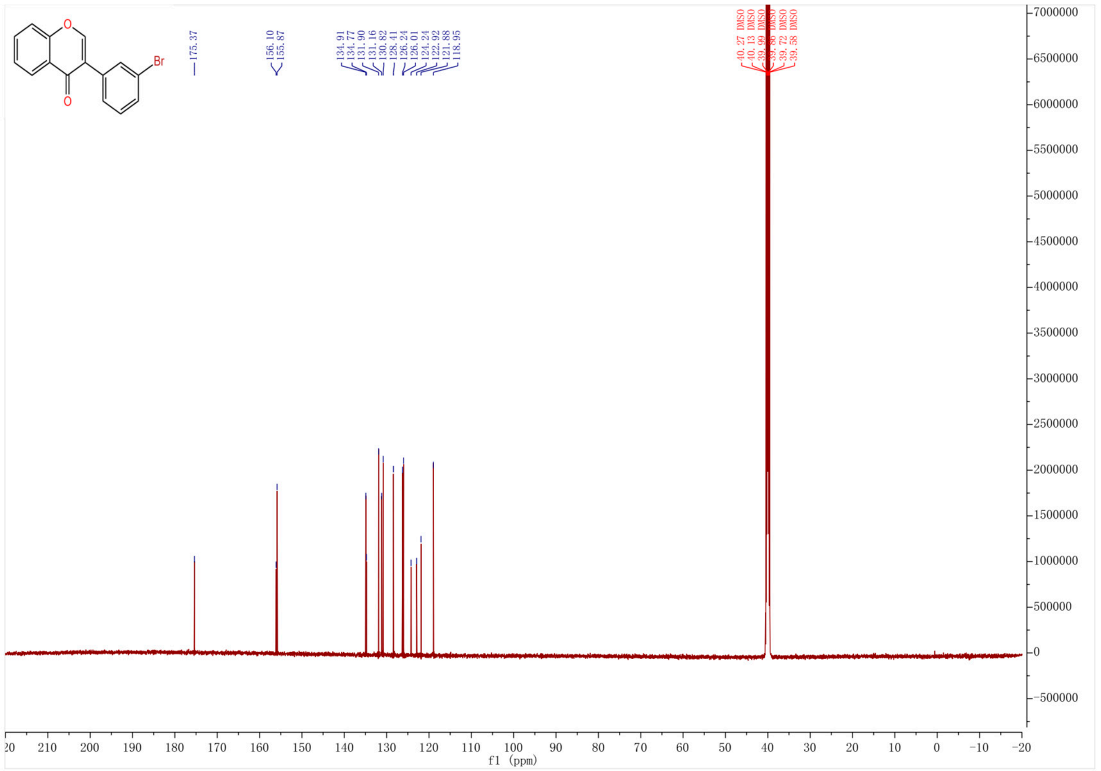

Figure S26. ^{13}C NMR spectra of compound **Z3**

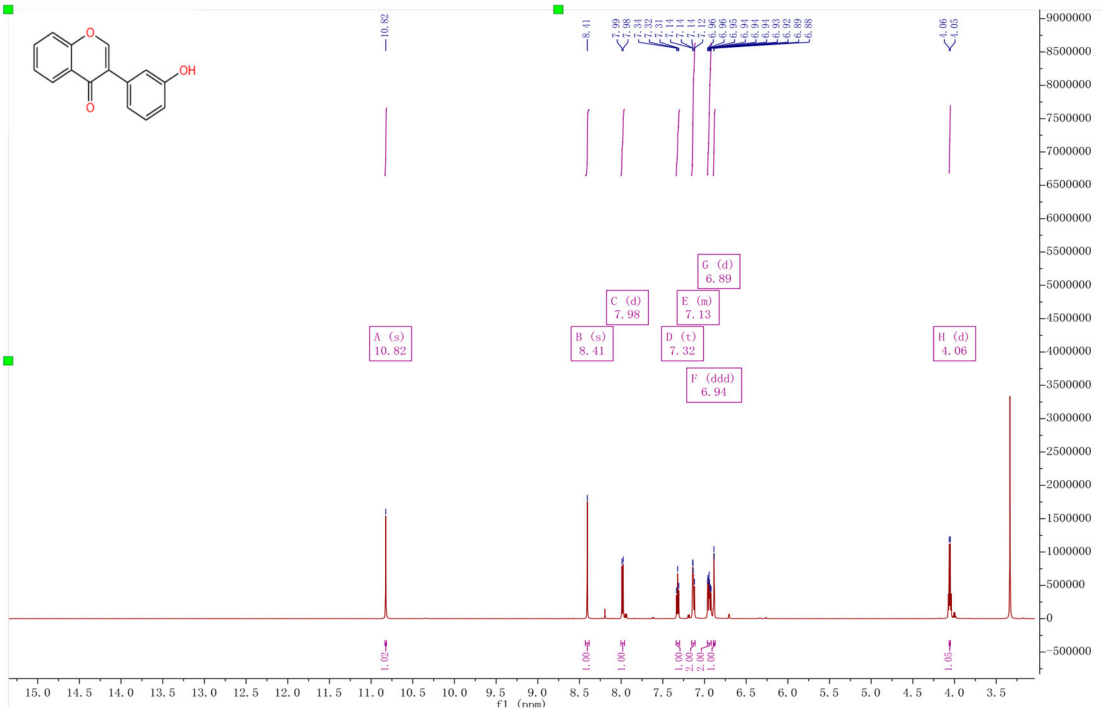

Figure S27.^{1}H NMR spectra of compound **Z5**

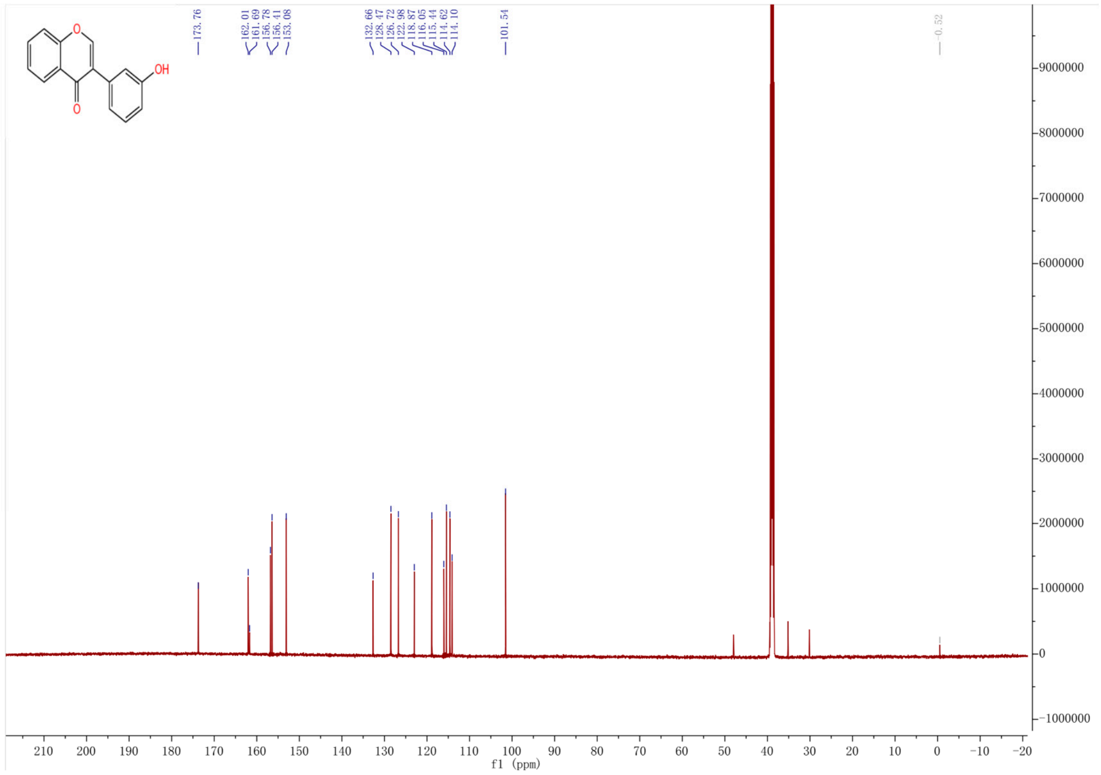

Figure S28. ^{13}C NMR spectra of compound **Z5**

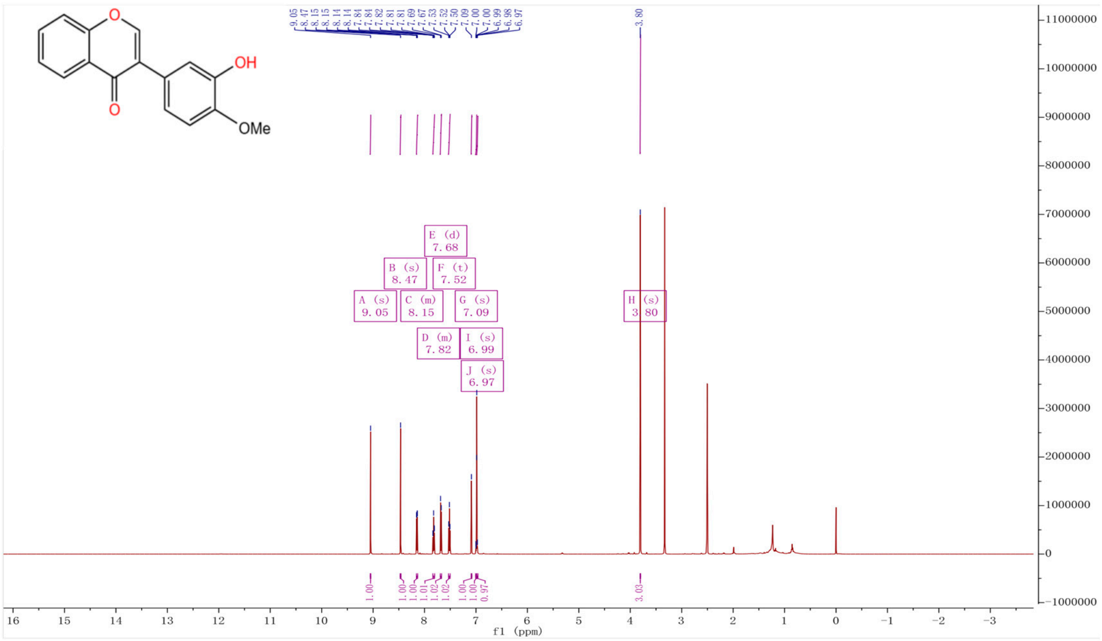

Figure S29.¹H NMR spectra of compound **Z6**

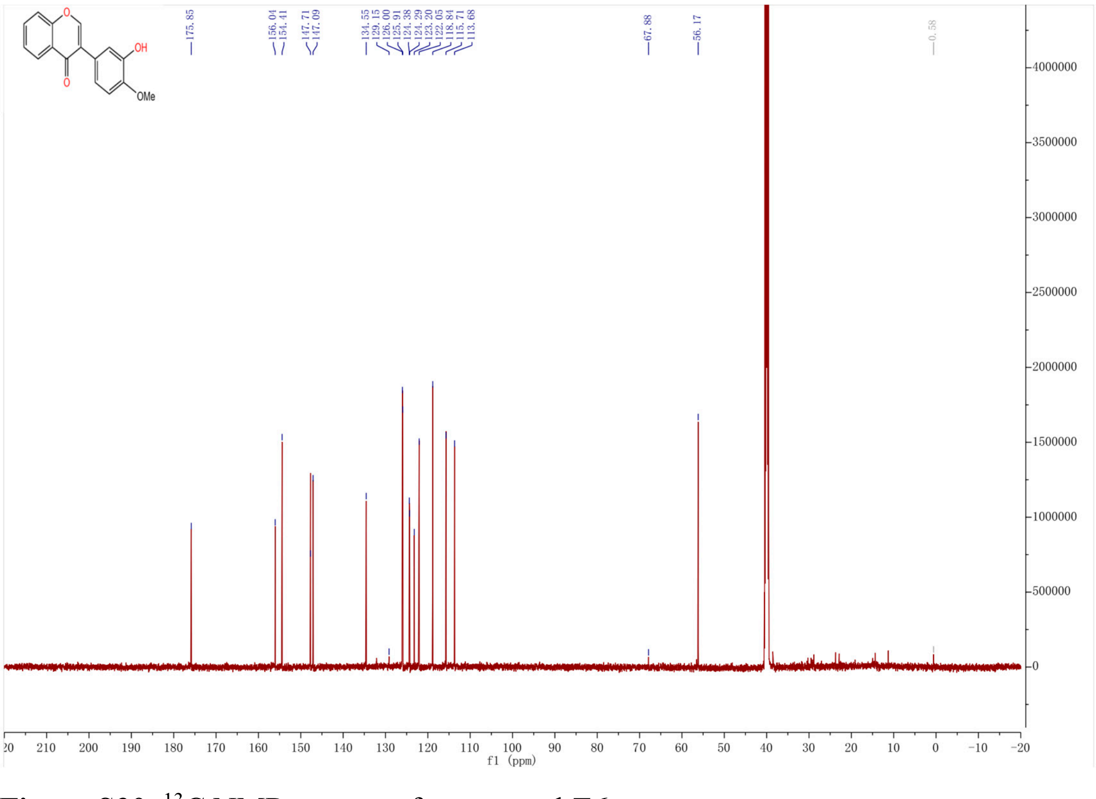

Figure S30. ^{13}C NMR spectra of compound **Z6**

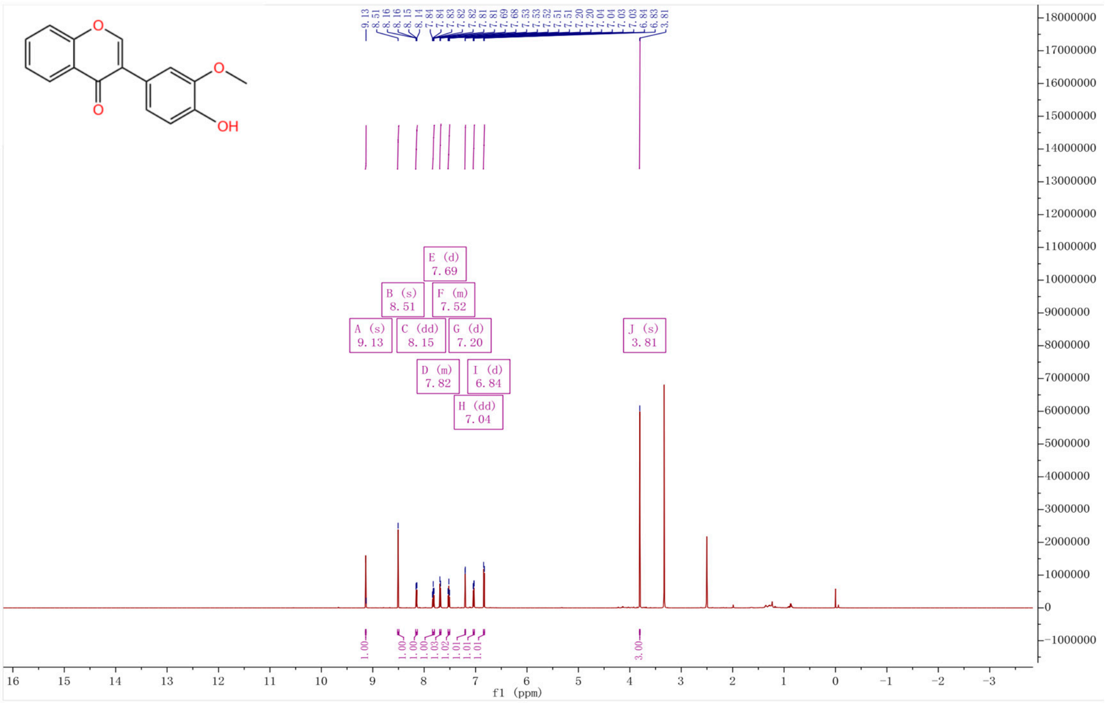

Figure S31.^{1}H NMR spectra of compound **Z7**

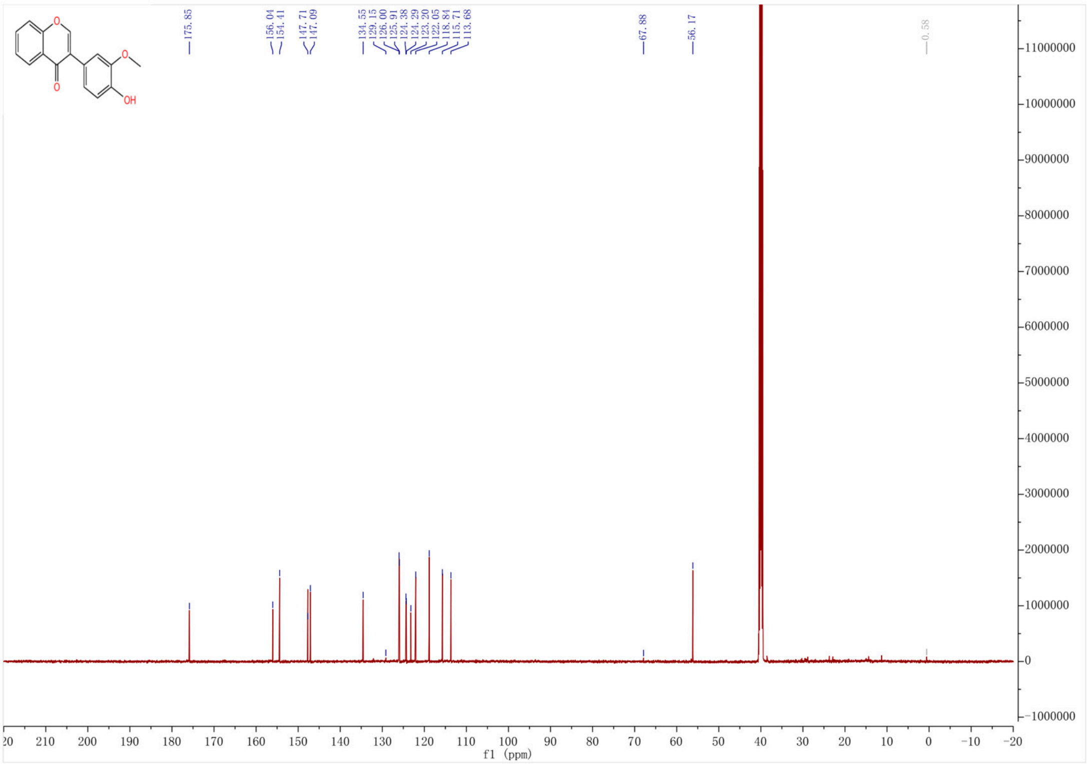

Figure S32. ^{13}C NMR spectra of compound **Z7**

EXPERIMENTAL

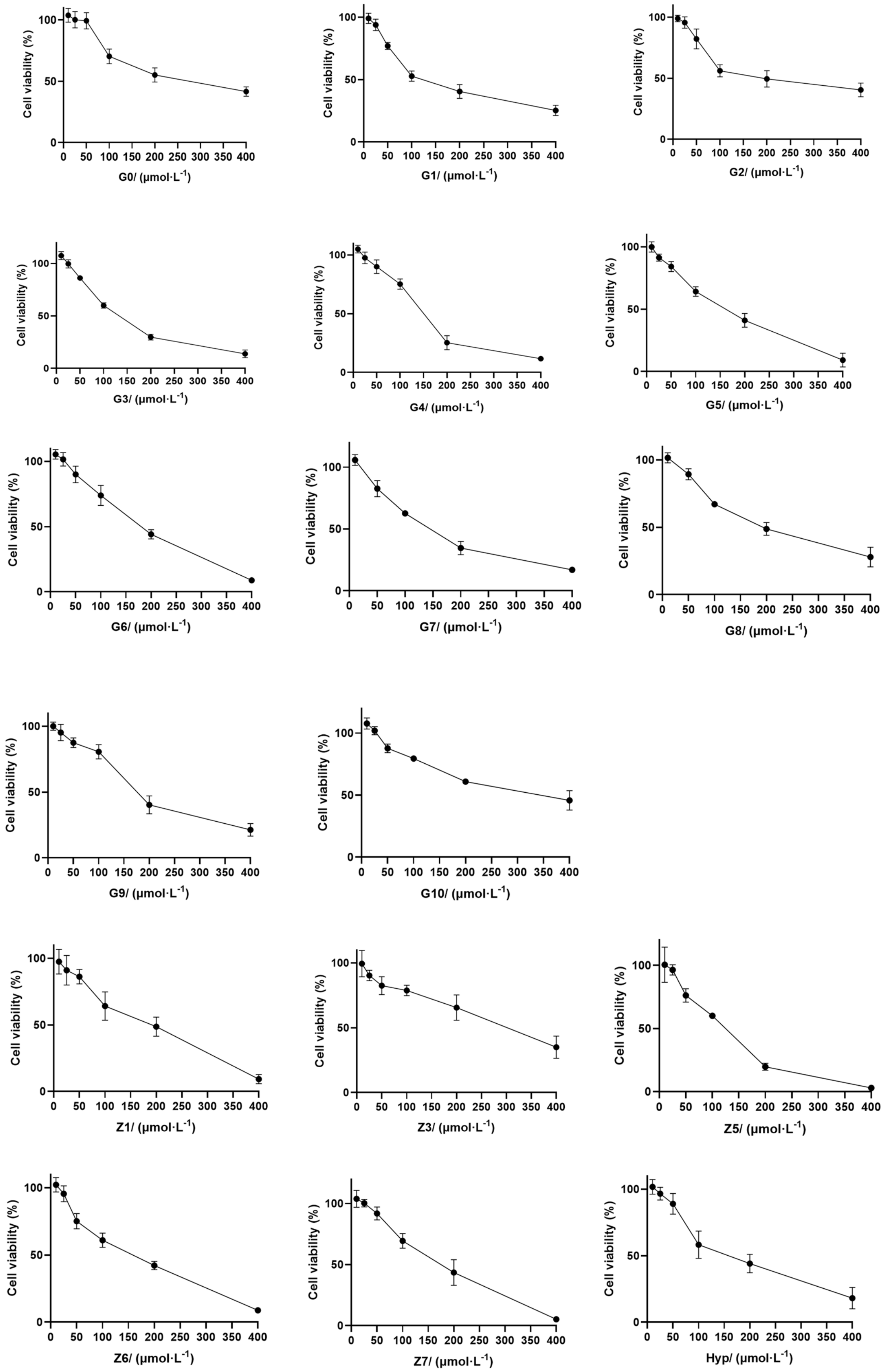

Figure S33 Effect of hypericin and its derivatives 48h on the activity of A549 cells
